# Aflibercept and Ang1 supplementation improve neoadjuvant or adjuvant chemotherapy in a preclinical model of resectable breast cancer

**DOI:** 10.1038/srep36694

**Published:** 2016-11-14

**Authors:** Florence T. H. Wu, Marta Paez-Ribes, Ping Xu, Shan Man, Elena Bogdanovic, Gavin Thurston, Robert S. Kerbel

**Affiliations:** 1 Department of Medical Biophysics, University of Toronto, Toronto, Ontario, Canada; 2 Biological Sciences Platform, Sunnybrook Research Institute, Toronto, Ontario, Canada; 3 Regeneron Pharmaceuticals, Inc., Tarrytown, New York, USA

## Abstract

Phase III clinical trials evaluating bevacizumab (an antibody to the angiogenic ligand, VEGF-A) in breast cancer have found improved responses in the presurgical neoadjuvant setting but no benefits in the postsurgical adjuvant setting. The objective of this study was to evaluate alternative antiangiogenic therapies, which target multiple VEGF family members or differentially modulate the Angiopoietin/Tie2 pathway, in a mouse model of resectable triple-negative breast cancer (TNBC). Neoadjuvant therapy experiments involved treating established orthotopic xenografts of an aggressive metastatic variant of the MDA-MB-231 human TNBC cell line, LM2-4. Adjuvant therapies were given after primary tumor resections to treat postsurgical regrowths and distant metastases. Aflibercept (‘VEGF Trap’, which neutralizes VEGF-A, VEGF-B and PlGF) showed greater efficacy than nesvacumab (an anti-Ang2 antibody) as an add-on to neoadjuvant/adjuvant chemotherapy. Concurrent inhibition of Ang1 and Ang2 signaling (through an antagonistic anti-Tie2 antibody) was not more efficacious than selective Ang2 inhibition. In contrast, short-term perioperative BowAng1 (a recombinant Ang1 variant) improved the efficacy of adjuvant chemotherapy. In conclusion, concurrent VEGF pathway inhibition is more likely than Ang/Tie2 pathway inhibition (e.g., anti-Ang2, anti-Ang2/Ang1, anti-Tie2) to improve neoadjuvant/adjuvant chemotherapies for TNBC. Short-term perioperative Ang1 supplementation may also have therapeutic potential in conjunction with adjuvant chemotherapy for TNBC.

The clinical utility of VEGF pathway-targeted antiangiogenic therapies is well-established in some cancer types. For instance, clinically approved antiangiogenic therapies for metastatic colorectal cancer include bevacizumab (an antibody against the VEGF-A ligand), aflibercept (a recombinant protein trap of the VEGF-A, VEGF-B and PlGF ligands) and ramucirumab (an antibody to VEGF receptor-2, VEGFR2) that are given with chemotherapy, as well as regorafenib monotherapy (a VEGFR2 tyrosine kinase inhibitor (TKI))^1^. For breast cancer, however, the clinical value of antiangiogenic therapy is still subject to ongoing debate and investigation^2^,^3^,^4^.

In the advanced metastatic setting of breast cancer (mBC), sunitinib (another VEGFR2 TKI) with or without chemotherapy failed to improve progression-free survival (PFS) in four phase III clinical trials^5^. With bevacizumab, results were mixed. In 2008, the FDA accelerated its approval of bevacizumab in the USA for HER2-negative (HER2^−^) mBC after a phase III trial (E2100^6^) showed a doubling of median PFS from 5.9 to 11.8 months when bevacizumab was added to first-line paclitaxel chemotherapy. But in 2011, FDA approval was revoked when subsequent phase III trials (AVADO^7^ and RIBBON-1^8^) showed much smaller PFS benefits (<3 months) when combining bevacizumab with other cytotoxic chemotherapy backbones. Nonetheless, bevacizumab with chemotherapy remains approved for mBC in Europe^1^. Overall survival (OS) benefits have never been observed in the five completed phase III trials which tested the addition of bevacizumab to first- or second-line chemotherapies for mBC (see Supplemental Table S1), although it still remains to be seen whether this will change with the maintenance or continuation of bevacizumab beyond disease progression^9^.

For early-stage non-metastatic HER2^−^ breast cancer in the preoperative (neoadjuvant) setting, bevacizumab consistently improved overall pathological complete response (pCR) rates when added to various cytotoxic chemotherapies in phase III clinical trials (GBG-44^10^, NSABP B-40^11^ and ARTemis^12^). Of the three trials, GBG-44 used the most stringent definition of pCR (see Supplemental Table S2), defined as the complete eradication of invasive disease in the breast and axillary lymph nodes plus non-invasive (intraductal) disease in the breast^10^. Using this definition, an improved pCR rate due to neoadjuvant bevacizumab therapy was observed only in the “triple-negative breast cancer (TNBC)” subgroup (i.e., HER2^−^ as well as negative for the estrogen receptor (ER) and progesterone receptor (PgR))^10^.

In the postoperative (adjuvant) setting of early-stage breast cancer, the addition of bevacizumab to adjuvant chemotherapies consistently failed to improve disease-free survival (DFS) in three phase III clinical trials regardless of breast cancer subtype (BEATRICE^13^, ECOG5103^14^, BETH^15^; see Supplemental Table S3). However, updated results from the NSABP B-40 trial showed an OS benefit associated with adding neoadjuvant-plus-adjuvant bevacizumab to standard neoadjuvant chemotherapies^16^.

As previously described, our lab has derived highly metastatic variants of the human breast carcinoma MDA-MB-231 cell line – including “LM2-4”^17^,^18^, “LM2-4^luc^”^19^ and “LM2-4^luc16^”^20^ – through consecutive cycles of orthotopic implantation, primary tumor resection, and isolation of spontaneous lung metastases. This “LM2-4 series” has proven to be a highly translational preclinical model of TNBC, through which we have recapitulated or predicted a number of the aforementioned clinical trial results with respect to antiangiogenic therapies, including: (i) the failure of sunitinib, with or without chemotherapy, in the advanced metastatic disease setting^21^; (ii) the efficacy of B20 and G6.31 (bevacizumab-like antibodies to VEGF-A) as neoadjuvant therapies^22^; and (iii) how the addition of DC101 (which, similar to bevacizumab, is an antibody-based antiangiogenic agent, but it targets VEGFR2 instead of VEGF-A) to paclitaxel chemotherapy yielded no benefit when this was restricted to adjuvant use but was effective when administered as a neoadjuvant-plus-adjuvant combination therapy^23^. The first objective of this present study was to extend this preclinical work by testing whether aflibercept may also have therapeutic potential, with or without paclitaxel, in the neoadjuvant and adjuvant settings of TNBC.

A second objective of this study relates to a newer class of investigational antiangiogenic drugs that target the Angiopoietin-Tie2 pathway. Within this class, trebananib (a bispecific peptibody against the Ang2 and Ang1 ligands) has failed two Phase III trials involving ovarian cancer^24^ and several Phase II trials including one that involved HER2^−^ mBC^25^. These setbacks have highlighted our incomplete understanding of how this complicated signaling pathway can be effectively targeted^24^. While Ang2 (a context-dependent Tie2 antagonist/partial agonist) has pro-angiogenic and vascular-destabilizing effects, Ang1 (a Tie2 receptor agonist) is an endogenous factor that limits vascular hyperpermeability and thus potentially a natural inhibitor of haematogenous metastatic dissemination^24^,^26^. Thus there is a growing view that selective neutralization of Ang2 might be superior to dual blockade of Ang2 and Ang1^24^. Extending this logic, direct Tie2 receptor inhibitors might similarly not be ideal, while Ang1 supplementation might actually have anti-metastatic potential. With this study, we directly compared these distinct Ang/Tie2 pathway-targeted strategies (anti-Ang2, anti-Tie2, versus Ang1 supplementation) head-to-head with VEGF pathway targeting (aflibercept) in the neoadjuvant and adjuvant settings of TNBC.

## Results

### Combining aflibercept vs. Ang/Tie2 pathway targeting with neoadjuvant chemotherapy

To model the preoperative neoadjuvant treatment setting, mice with established orthotopic primary LM2-4 tumors around 150 mm^3^ in volume were randomized and treated for 2 weeks with either the controls, aflibercept (which neutralizes VEGF-A, VEGF-B and PlGF), nesvacumab (an antibody to Ang2), BowAng1 (a recombinant Ang1 variant), or an anti-Tie2 antibody, with or without paclitaxel chemotherapy. Response to therapy was assessed by clinically-relevant parameters^27^ – reductions in primary tumor burden, tumor vascularity, and tumor invasiveness.

**Figure 1:**
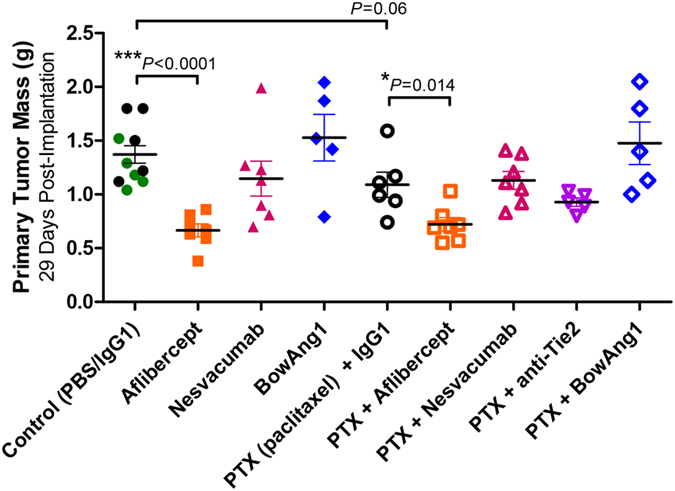
Aflibercept is more potent than paclitaxel chemotherapy or Ang/Tie2-targeted agents in terms of inhibiting primary breast tumor growth. 14 days after orthotopic implantation of 2 × 10^6^ LM2-4 cells, mice bearing ~150-mm^3^ primary breast tumors were randomized and administered with either the controls (PBS vehicle or IgG1 isotype), aflibercept (anti-VEGF-A/VEGF-B/PlGF), nesvacumab (anti-Ang2), BowAng1, or an anti-Tie2 antibody, with or without paclitaxel chemotherapy, for 2 weeks. End-point tumor mass, in grams, is plotted with mean ± SEM depicted. Predefined comparisons were subjected to two-sampled unpaired *t* tests (n = 5 to 11). Mice given PBS vehicle alone (green solid circles) versus a non-specific IgG1 antibody (black solid circles) did not have significantly different mean terminal tumor weights (*P* = 0.11); these mice were considered as a single ‘untreated control’ group (n = 11) in subsequent analyses for treatment effects.

First, we assessed residual primary tumor burden. Compared to untreated controls, the only monotherapy that significantly reduced terminal tumor mass was aflibercept (52% reduction; *P* < 0.0001; 95% CI of 34% to 69%; Fig. 1). Compared to paclitaxel alone, the only combination therapy that led to a significant further reduction in terminal tumor mass was with concurrent aflibercept (34% reduction; *P* = 0.014; 95% CI of 8% to 60%; Fig. 1). Thus, VEGF targeting was particularly effective at restricting primary tumor growth; in contrast, the Ang/Tie2 pathway-targeted agents (anti-Tie2, anti-Ang2, BowAng1) were relatively ineffective at controlling primary tumor growth (Fig. 1 and Supplemental Fig. S1).

**Figure 2:**
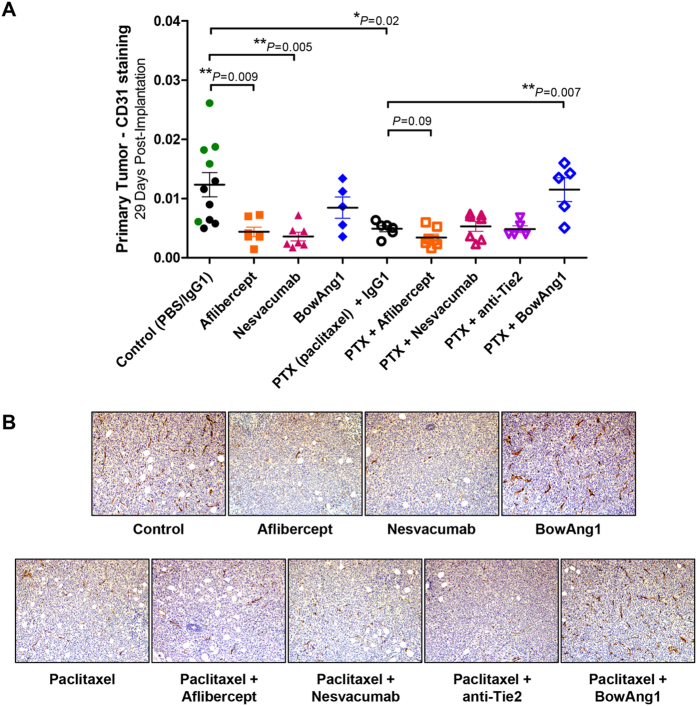
Differential treatment effects on primary breast tumor blood vessels. Orthotopic primary LM2-4 breast tumors harvested after 2 weeks of therapy were analyzed for tumor vascularity by CD31 staining. (**A**) Automated quantification of CD31-positive pixels normalized to total pixels at 100×, with mean ± SEM depicted. Predefined comparisons (control vs. monotherapies; paclitaxel monotherapy vs. paclitaxel-containing combinations) were subjected to two-sampled unpaired *t* tests (n = 5 to 11 mice per group). (**B**) Representative microscopy images of CD31-stained breast tumor sections.

Next, we assessed primary tumor vascularity via immunohistochemistry staining of CD31 (PECAM-1), a commonly used marker of endothelial cells. As monotherapies, aflibercept, nesvacumab, and paclitaxel all effectively reduced CD31 positivity in primary LM2-4 breast tumors (Fig. 2A, *P* < 0.05). In contrast, single-agent BowAng1 had no significant effect on CD31 positivity, while concurrently administered BowAng1 significantly increased CD31 positivity compared to paclitaxel treatment alone (Fig. 2A, *P* = 0.007). This potentially indicates a stabilization of tumor blood vessels after 2 weeks of BowAng1 therapy, albeit insufficient to significantly promote primary tumor growth. No statistically significant increases in primary tumor burden were observed with BowAng1 either as a single agent or when combined with paclitaxel (Fig. 1 and Supplemental Fig. S1; *P* > 0.10). In a separate experiment testing a different engineered recombinant Ang1 variant, COMP-Ang1 treatment also only resulted in a trend of slightly larger orthotopic primary LM2-4 tumors (Fig. S2A, *P* = 0.11).

**Figure 3:**
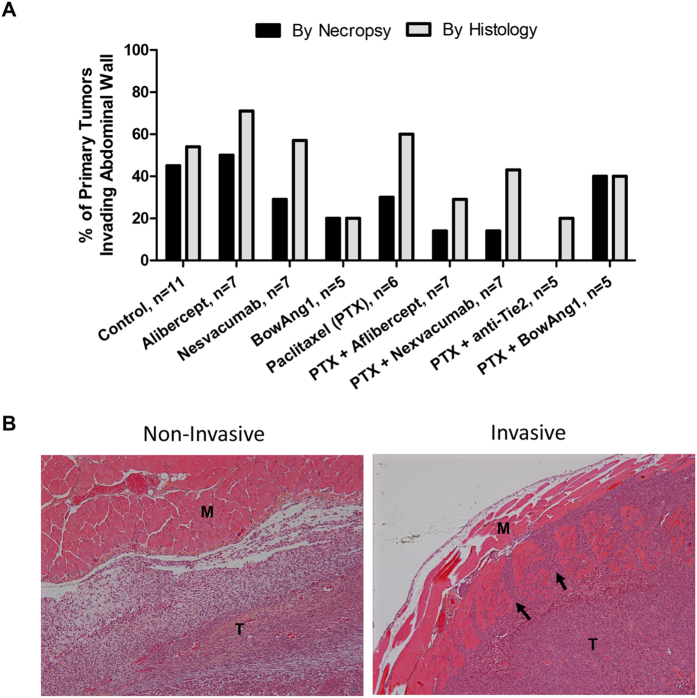
Differential treatment effects on primary breast tumor invasiveness into the abdominal wall. 14 days after orthotopic implantation of 2 × 10^6^ LM2-4 cells, mice bearing ~150-mm^3^ primary breast tumors were randomized and administered with either the controls (PBS vehicle or IgG1 isotype), aflibercept (anti-VEGF-A/VEGF-B/PlGF), nesvacumab (anti-Ang2), BowAng1, or an anti-Tie2 antibody, with or without paclitaxel chemotherapy, for 2 weeks. On day 29 post-implantation, all mice were sacrificed and their primary breast tumors were examined during necropsy and confirmed histologically for signs of invasion into the abdominal wall. (**A**) The incidence (%) of invaded tumors per treatment group is plotted. *P* > 0.05 by Fisher’s exact test. (**B**) Representative microscopy images from the histological analysis of primary LM2-4 breast tumors for invasions into the adjacent abdominal wall by hematoxylin and eosin staining. “M” denotes abdominal wall muscle. “T” denotes tumor cells. Black arrows mark regions where tumor cells are infiltrating into the abdominal wall and separating muscular fascicles.

Primary tumor invasiveness – specifically, infiltrations from the mammary fat pad into the adjacent abdominal wall – was assessed by gross examination during necropsy as well as by histology. Among the monotherapies, BowAng1 showed the greatest potential for inhibiting tumor invasiveness compared to untreated controls – histology revealed a decrease from 54% to 20% (Fig. 3A). We should note that similar results were observed in a separate experiment with COMP-Ang1 therapy reducing LM2-4 tumor invasiveness from 52% to 31% (Supplemental Fig. S2B). Among the combination therapies, concurrent anti-Tie2 showed the greatest potential for inhibiting tumor invasiveness compared to paclitaxel alone – histology revealed a decrease from 50% to 20% (Fig. 3A). Interestingly, aflibercept as a monotherapy showed trends of increasing local invasions, but when added to paclitaxel, it showed the opposite trends of suppressing local invasions (Fig. 3A). This is consistent with our recently published finding whereby in four different TNBC xenograft models (three cell lines, MDA-MB-231, MDA-MB-468 and MDA-MB-435, as well as a patient-derived xenograft model, HCI-002), antiangiogenic DC101 monotherapy (VEGFR2 blockade) had pro-invasive effects which were blocked by concurrent chemotherapy (paclitaxel or cyclophosphamide)^23^.

Lastly, microscopic lung metastases were not visible by gross examination at the time of necropsy. Histological examination confirmed the sparsity of lung micrometastases and no therapy-associated differences could be discerned (Supplemental Fig. S3).

### Combining aflibercept vs. Ang/Tie2 pathway targeting with adjuvant chemotherapy

To model the postoperative adjuvant treatment setting, mastectomy of the right inguinal mammary fat pad was performed on mice to resect established primary LM2-4 tumors.

**Figure 4:**
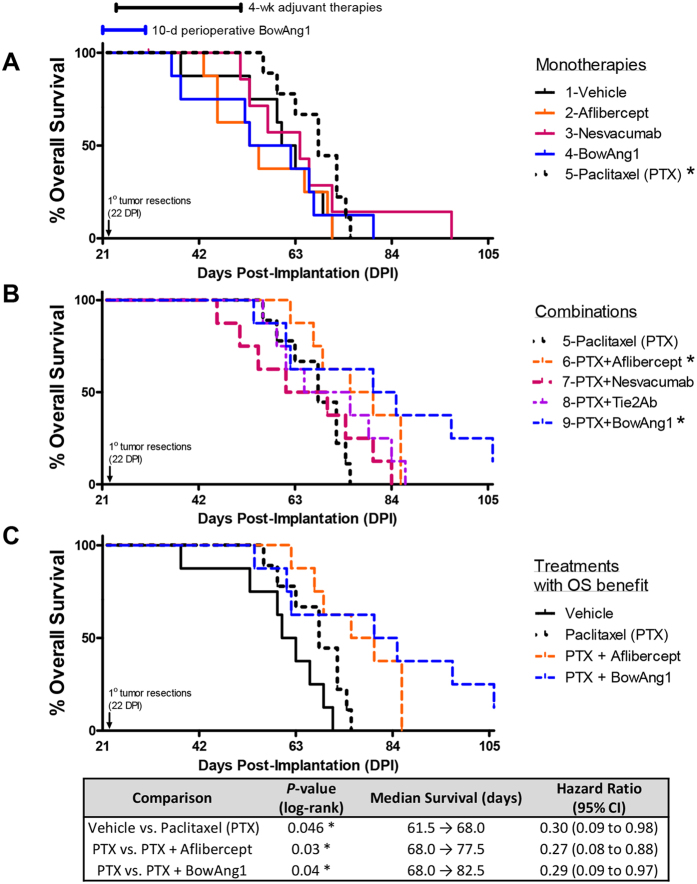
Addition of adjuvant aflibercept or perioperative BowAng1 improves adjuvant paclitaxel chemotherapy for resected breast cancer. 18 days after orthotopic implantation of 2 × 10^6^ LM2-4 cells, mice bearing roughly 400-mm^3^ primary breast tumors were randomized into nine treatment groups. Primary tumor resections by complete mastectomies were performed at 22 days post-implantation (DPI). BowAng1 was given as a 10-day perioperative therapy beginning one day before surgery (21 DPI). Aflibercept (anti-VEGF-A/VEGF-B/PlGF), nesvacumab (anti-Ang2), the anti-Tie2 antibody, paclitaxel chemotherapy, and combinations thereof, were given as 4-week-long adjuvant therapies starting two days after surgery (24 DPI). Dosing schedule is depicted above survival curves. N = 7–9 mice per treatment group. Kaplan-Meier analyses of overall survival: (**A**) comparison of monotherapy groups; (**B**) comparison of combination therapies; and (**C**) showing only those single-agent and combination therapies that led to a statistically significant overall survival benefit compared to vehicle control and chemotherapy alone respectively (*P* < 0.05, log-rank test).

In the first adjuvant therapy experiment (Fig. 4), primary tumors ≥400 mm^3^ in size were resected on day 22 post-implantation of 2 × 10^6^ LM2-4 cells. Perioperative BowAng1 was given as a 10-day perioperative therapy, beginning one day before resection. Adjuvant aflibercept, nesvacumab, the anti-Tie2 antibody, paclitaxel chemotherapy, and combinations thereof, were given as 4-week-long adjuvant therapies beginning two days after resection. While the majority of mice reached endpoint with labored breathing (due to lung metastases) and/or limb paralysis (due to large axillary, brachial or inguinal lymph node metastases), about half of the mice also developed local regrowths at the primary tumor site and/or ascites (51% and 24% respectively, Supplemental Table S4). Kaplan-Meier analysis of overall survival (OS) revealed that among the adjuvant monotherapies tested (Fig. 4A), only paclitaxel (PTX) led to an OS benefit (*P* = 0.046, HR = 0.30). The lack of efficacy of perioperative BowAng1 as a single-agent (Fig. 4A) was reproduced with COMP-Ang1 in a separate experiment (Supplemental Fig. S2C). However, among the combination therapies tested (Fig. 4B,C), the OS benefit of adjuvant PTX chemotherapy was further improved by the addition of aflibercept (*P* = 0.03, HR = 0.27) or BowAng1 (*P* = 0.04, HR = 0.29). Concurrent perioperative BowAng1 led to trends of reduced invasive primary tumor regrowths and ascites when compared to adjuvant PTX alone (Supplemental Table S5). In contrast, no OS benefits were observed with the addition of nesvacumab or anti-Tie2 to PTX (Fig. 4B).

**Figure 5:**
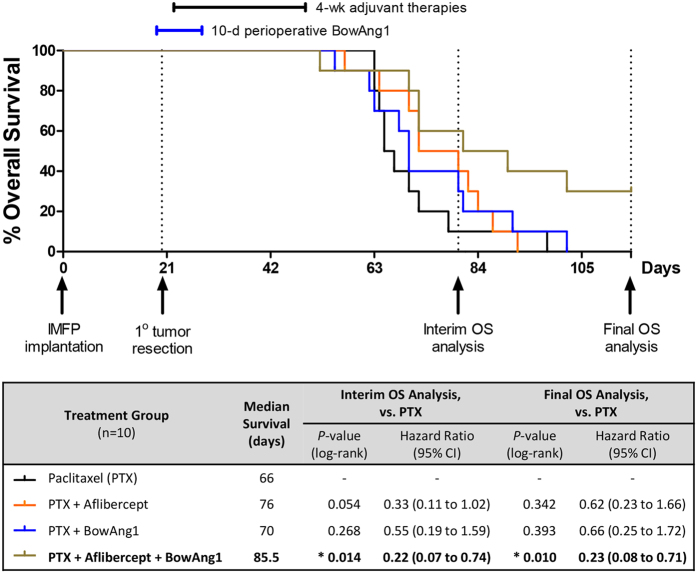
Triple combination of adjuvant aflibercept, perioperative BowAng1 and adjuvant paclitaxel therapies for resected breast cancer. 18 days after orthotopic implantation of 2 × 10^6^ LM2-4 cells, mice bearing approximately 200-mm^3^ primary breast tumors were randomized into four treatment groups. Primary tumor resections by complete mastectomies were performed at 20 days post-implantation (DPI). BowAng1 was given as a 10-day perioperative therapy, beginning one day before surgery (19 DPI). Aflibercept (anti-VEGF-A/VEGF-B/PlGF) and paclitaxel chemotherapy were given as 4-week-long adjuvant therapies, starting two days after surgery (22 DPI). Dosing schedule is depicted above Kaplan-Meier survival curves (n = 10 mice per treatment group). The log-rank statistical test was used to assess differences in overall survival between treatment groups at two timepoints: 80 DPI (interim) and 115 DPI (final) respectively.

A second adjuvant therapy experiment (Fig. 5) was performed to validate the most promising combinations identified above and to additionally test the triple combination of PTX plus aflibercept plus BowAng1. This time, primary tumors ≥200 mm^3^ in size were resected on day 20 post-implantation, earlier than in the previous experiment, in order to lower the incidence of local tumor regrowths and ascites by endpoint (to 18% and 10% overall respectively, Supplemental Table S5). As a result, mortality was predominantly due to metastatic burden in the lungs or distant lymphatics (Supplemental Table S5). As before, BowAng1 was given as a 10-day perioperative therapy beginning one day before resection, while aflibercept and paclitaxel were given as 4-week-long adjuvant therapies beginning two days after resection. While the doublets, PTX + BowAng1 and PTX + aflibercept, again showed trends of prolonging OS compared to PTX alone, these improvements were not statistically significant at the time of final analysis (Fig. 5). The seemingly lesser efficacy of these doublets compared to the previous experiment (Fig. 4C) could potentially be related to the reduced incidence of local regrowths at the primary tumor site, which potentially may be more responsive to these therapies than the lung and lymphatic metastases. Interestingly, the PTX + aflibercept doublet had been associated with a greater OS benefit during interim analysis (Fig. 5, *P* = 0.054, HR = 0.33) than was apparent from the final analysis (Fig. 5, *P* = 0.342, HR = 0.62) – which is reminiscent of clinical trial observations where the initial DFS advantages associated with adjuvant use of antiangiogenic drugs (bevacizumab in colon and breast cancer trials^13^,^28^,^29^ and sunitinib/sorafenib in a renal cell carcinoma trial^30^) faded over time after cessation of these adjuvant therapies. In this experiment, the triple combination of PTX + aflibercept + BowAng1 proved to be the most effective at prolonging OS (Fig. 5, *P* = 0.01, HR = 0.23). While aflibercept and BowAng1 combined well in the presence of concurrent paclitaxel (Fig. 5), this was not the case with sunitinib plus COMP-Ang1 in a separate experiment (Supplemental Fig. S2D).

## Discussion

Our major findings from the spontaneously metastasizing LM2-4 model of human TNBC were as follows. First, aflibercept (a recombinant protein that neutralizes three members of the VEGF family of ligands, VEGF-A, VEGF-B and PlGF) showed greater therapeutic potential than nesvacumab (Ang2 inhibition) as an add-on to neoadjuvant and adjuvant paclitaxel chemotherapy. Second, concurrent inhibition of Ang1 and Ang2 signaling via Tie2 (through an antagonistic Tie2 antibody) did not confer a therapeutic advantage over selective inhibition of Ang2 (through nesvacumab) in the adjuvant setting. Third, on the contrary, Ang1 supplementation (through BowAng1) during a short perioperative window improved the efficacy of adjuvant paclitaxel chemotherapy, with or without aflibercept. In the paragraphs below, we elaborate on the basis for these conclusions.

In a recent preclinical study by Paez-Ribes *et al*., we had shown in the resected orthotopic LM2-4 breast cancer model that DC101 (VEGFR2-specific inhibition) was unable to improve OS when added to adjuvant paclitaxel chemotherapy^23^. This preclinical finding mirrored clinical trial observations where bevacizumab (VEGF-A-specific inhibition) had also failed to improve DFS when added to adjuvant chemotherapies^13^,^14^,^15^. Using the same resected LM2-4 TNBC model, we report in this current study that, unlike DC101, aflibercept was able to significantly improve OS when combined with adjuvant paclitaxel (Fig. 4D). The apparent advantage of aflibercept over DC101 could be a reflection of the fact that DC101 only inhibits VEGFR2 signaling (which is mainly mediated by VEGF-A), while aflibercept additionally inhibits VEGF-B/PlGF-mediated VEGFR1 signaling^31^,^32^,^33^,^34^.

Moreover, we also report in the unresected LM2-4 model that the addition of aflibercept to paclitaxel enhanced primary tumor growth inhibition, which suggests therapeutic potential in the neoadjuvant setting as well. Previously, Paez-Ribes *et al*. had shown in the LM2-4 model an OS advantage of administering DC101 + PTX as a neoadjuvant-plus-adjuvant regimen rather than restricting its delivery to the adjuvant setting^23^. In line with this preclinical result, a phase III clinical trial (NSABP-B-40) subsequently reported an OS benefit associated with the combination of neoadjuvant-plus-adjuvant bevacizumab with standard neoadjuvant chemotherapies for breast cancer as a secondary outcome^16^. Thus, like bevacizumab and DC101, aflibercept may be yet another VEGF pathway-targeted antiangiogenic agent worth further testing in combination with neoadjuvant-plus-adjuvant chemotherapy regimens, at least in TNBC.

As alluded to in the introduction, phase II evaluation of trebananib (AMG386, a bispecific peptibody against Ang2 and Ang1) in the advanced metastatic setting of HER2^−^ breast cancer showed no PFS benefit by adding trebananib to paclitaxel chemotherapy, with or without bevacizumab^25^. No Ang2 inhibitors have yet been evaluated in TNBC clinical trials in the neoadjuvant or adjuvant settings.

Our data from the unresected and resected LM2-4 model suggests the inferiority of nesvacumab (Ang2 neutralization) compared to aflibercept (VEGF-A, VEGF-B and PlGF neutralization), whether as single agents or as add-ons to paclitaxel chemotherapy, in both the neoadjuvant and adjuvant settings of TNBC. Furthermore, an antagonistic antibody to Tie2 (REGN1376) also failed to improve adjuvant paclitaxel chemotherapy in our resected LM2-4 model. This antibody – which blocks the binding of both Ang1 and Ang2 to the Tie2 receptor – functionally approximates, to some extent, dual Ang1/Ang2-targeted agents like trebananib. The fact that REGN1376 did not yield greater efficacy than nesvacumab in our LM2-4 model as well as other tumor models^35^ suggest that simultaneous Ang1 inhibition often does not confer a therapeutic advantage over selective inhibition of Ang2. Altogether, this preclinical study predicts limited capacity of anti-Ang2, anti-Ang1/2, and antagonistic anti-Tie2 agents as add-ons to neoadjuvant and adjuvant chemotherapies for TNBC. Unexpectedly, the only Ang/Tie2 pathway-targeted therapy found to improve the efficacy of paclitaxel chemotherapy in our models was the Tie2 agonist, BowAng1 – when used in a certain manner as explained further below.

Our results with respect to the nesvacumab – specifically its inability to improve adjuvant paclitaxel (PTX) chemotherapy in the resected LM2-4 model – may appear to contradict the recent preclinical findings of Srivastava *et al*.^36^, at least superficially until the following details are considered. Srivastava *et al*. had used Abraxane^TM^, the albumin-bound nanoparticle formulation of PTX, with inherently different pharmacokinetic and toxicity profiles compared to the standard PTX used in our current study. Using a resected 4T1 murine breast cancer model, Srivastava *et al*. had tested a different anti-Ang2 agent (murine-chimeric LC06; Roche), which was found to improve the efficacy adjuvant PTX chemotherapy when Abraxane^TM^ is given at low-doses in a metronomic fashion (6 mg/kg, IP, qd, long-term) but not when Abraxane^TM^ is given at conventional maximum tolerated doses (MTD: 30 mg/kg, IP, qdx5, 1 cycle). In our current study, standard PTX was dosed as an MTD regimen (30 mg/kg, IP, q2w). We did not test a metronomic PTX regimen.

Conceptually, there are pros and cons associated with Ang1 supplementation in the context of cancer treatment, depending on whether normal (host) blood vessels or tumor blood vessels are targeted. The potential benefit stems from the fact that Ang1 is an endogenous vascular-stabilizing factor. Its anti-permeability and anti-inflammatory effects on the ‘normal’ host vasculature should theoretically limit tumor cell extravasation and vessel co-option at distant organs – in essence, allowing Ang1 to counteract some of the secondary pro-metastatic mechanisms of VEGF and Ang2^37^,^38^,^39^,^40^,^41^. The potential risk is that Ang1 might also act as a stabilizing, protective, or maturation factor for angiogenic tumor blood vessels – hijacked into being a cooperative partner of VEGF and Ang2 to promote tumor growth and/or facilitate metastasis^26^.

In the published literature, genetic overexpression of Ang1 has led to tumor growth suppression in some preclinical models^42^,^43^,^44^,^45^ but has also promoted tumor growth or metastasis in others^46^,^47^,^48^. Delivering Ang1 in protein form is potentially a more clinically feasible approach. In one preclinical study, subcutaneous administration of BowAng1 protein alone did not affect tumor growth, but in combination completely blocked the anti-tumor and anti-angiogenic activity of nesvacumab in subcutaneously implanted Colo205 colorectal and A431 epidermoid primary tumors^49^. In another preclinical study, subcutaneous injections of BowAng1 protein – in the long term (20–27 days), but not in the short term (5 days) – interfered with the anti-tumor activity of aflibercept in intra-renally implanted SK-NEP-1 primary tumors^50^. Neither of these studies involved actual surgical resection of primary tumors to model the adjuvant/perioperative use of Ang1 supplementation in a clinically-relevant manner.

Using a clinically-relevant model of resected TNBC in our present study, we have restricted Ang1 supplementation therapy to a relatively narrow ‘perioperative window’. BowAng1 was given as a 10-day therapy beginning one day prior to surgical resections of orthotopic primary tumors. Alternatively, COMP-Ang1 (a recombinant pentameric Ang1 variant) was given as a 12-day therapy beginning 4 days prior to surgical resections. These designs were intended to minimize the potential risks of exposing angiogenic tumor blood vessels to exogenous Ang1 (i.e., minimizing the preoperative exposure of primary tumors and postoperative exposure of tumor regrowths or metastases) while maximizing the potential benefits of targeting the ‘normal’ host vasculature (i.e., to impede distant metastatic seeding).

In the resected LM2-4 breast cancer model, we observed that the addition of perioperative BowAng1 to adjuvant paclitaxel chemotherapy can significantly improve OS, at least in part by lowering the incidence of invasive local tumor regrowths as well as ascites. We should emphasize that OS benefits were only observed when perioperative BowAng1 was used in combination with paclitaxel chemotherapy, but not when BowAng1 or COMP-Ang1 was used as a single agent, and also not when COMP-Ang1 was combined with adjuvant sunitinib therapy.

Our finding that short-term perioperative Ang1 supplementation may suppress the invasiveness of postsurgical tumor regrowths is promising and suggests that further investigation is warranted, but with special considerations. Our data stresses the importance of restricting the duration of Ang1 supplementation. Longer treatments of established primary breast tumors with either BowAng1 or COMP-Ang1 had led to slight trends of larger tumor volumes despite simultaneous trends of reduced tumor invasiveness. Interestingly, this appears to be the reverse scenario of how VEGF/VEGFR2 pathway inhibitors can be simultaneously associated with tumor growth inhibition and aggravated invasiveness^51^. A slight increase in tumor size after Ang1 supplementation therapy, even when not detrimental to OS in the long term, could nevertheless be alarming to patients and oncologists, registering as “disease progression” or “treatment failure” when treatment response is evaluated by the conventional RECIST criteria used in clinical trials.

## Methods

### Orthotopic breast cancer xenografts

*In vivo* experiments were approved by the Sunnybrook Research Institute Animal Care Committee and carried out in strict accordance with the Canadian Council of Animal Care guidelines. All surgical and terminal procedures were performed under inhaled isoflurane anesthesia. Buprenorphine was given subcutaneously (SC) as pre- and post-operative analgesia. The LM2-4 cell line is an aggressively metastatic derivative of the MDA-MB-231 human breast cancer cell line^17^,^18^ that is periodically authenticated and subjected to mycoplasma screening as previously described^23^. LM2-4 cells were cultured in DMEM High Glucose media supplemented with 5% fetal bovine serum, in humidified incubators (37 °C, 21% O_2_, 5% CO_2_), and harvested at 80% confluence into single-cell suspensions. Orthotopic implantations involved injecting 2 × 10^6^ LM2-4 cells suspended in 50 μL of serum-free media into the right inguinal mammary fat pad of 6 to 8-week-old female CB-17 SCID mice from Charles River Canada. Mammary tumor volumes were serially tracked by caliper measurements (0.5 × width^2^ × length). Endpoint criteria for survival experiments in the adjuvant therapy setting included: labored breathing (lung metastases); ascites (tumor invasions into the abdominal cavity); primary tumor regrowths or lymphatic metastases reaching endpoint volumes (1500 mm^3^) or causing limb paralysis/immobility; and 20% weight loss.

### *In vivo* treatments

**Aflibercept** (Regeneron Pharmaceuticals) – a recombinant fusion protein combining the second Ig (VEGF-A/VEGF-B/PlGF-binding) domain of human VEGFR1, the third Ig (VEGF-A-binding) domain of human VEGFR2, and the Fc region of the human IgG1 antibody^31^,^52^ – was administered at 5 mg/kg, 2x/wk, SC. **Nesvacumab** (REGN910; Regeneron) – a fully human IgG1 monoclonal antibody that binds both human and murine Ang2 with high affinity, but not Ang1^49^,^53^ – was administered at 5 mg/kg, 2x/wk, SC. **BowAng1** (REGN108, also known as Ang-F1-Fc-F1 or Ang1-Fd-Fc-Fd; Regeneron) – an engineered variant of tetrameric human Ang1, made by the recombinant fusion of four fibrinogen-like (receptor-binding) domains from Ang1 to a dimer of human IgG1 Fc domains^49^,^54^ – was administered at 25 mg/kg, 3x/wk, SC. The **Tie2 antibody** (REGN1376; Regeneron) – which antagonistically binds Tie2 at its ligand-binding site^35^ – was administered at 10 mg/kg, 2x/wk, SC. **Paclitaxel** (DIN: 02391465; Accord Healthcare Inc.) was administered at 30 mg/kg, every 2 weeks, intraperitoneally (IP). All drugs were diluted in phosphate-buffered saline (PBS), which hence served as the vehicle control (IP or SC). A human IgG1 antibody with no binding to mouse or human proteins (REGN1945; Regeneron; administered at 5 mg/kg, 2x/wk, SC) served as an additional control.

### Histological analyses

Surgically-dissected primary breast tumors (with adjacent sections of abdominal wall) and lungs were fixed in 10% buffered formalin overnight and stored in 70% ethanol before paraffin-embedding. To determine the incidence of invaded primary tumors per treatment group, serial 5 μm-thick sections (5 sections per animal, taken 50 μm apart along the breast tumor-abdominal wall boundary) were subjected to standard hematoxylin and eosin (H&E) staining. To assess primary tumor vascularity, serial 5 μm-thick sections (2–4 sections per animal, >150 μm apart) were subjected to immunohistochemistry (IHC) staining for murine CD31: a boiling sodium citrate buffer (10 mM, pH 6.0) was used for antigen retrieval; 1% hydrogen peroxide (15 mins) was used for quenching of endogenous peroxidases; 10% rabbit serum in protein block (DAKO #X0909) was used to reduce non-specific binding; a rat anti-mouse CD31 primary antibody (clone SZ31, Dianova #DIA-310, 1:50) in diluent (DAKO #S3022) was applied at 4 °C overnight; a biotinylated rabbit anti-rat IgG secondary antibody (Jackson Immunoresearch; 1:200) in diluent (DAKO #S3022) was applied at room temperature for 30 mins; detection involved an ABC-HRP kit (VECTASTAIN Elite), a DAB + chromogen-substrate system (DAKO #K3467), and hematoxylin counterstaining; ImageJ software was used for color deconvolution of microscopy images and automated quantification of CD31^+^ pixels normalized to total pixels (6–8 fields of view analyzed per section, at 100× magnification, using a Leica DM LB2 microscope and DFC 300 FX camera). To assess pulmonary metastases, 5 μm-thick sections of lung tissue were subjected to IHC staining for human vimentin, using a similar protocol as above, except with a murine anti-human vimentin primary antibody (Invitrogen #18-0052, clone V9, 1:100), donkey serum for blocking, and a universal kit containing biotinylated anti-mouse/rabbit secondary antibodies and streptavidin-HRP for detection (DAKO# K0690).

### Statistical analysis

GraphPad Prism software (San Diego, USA) was used for statistical analysis. See figure legends for specific statistical tests used.

## Additional Information

**How to cite this article**: Wu, F. T. H. *et al*. Aflibercept and Ang1 supplementation improve neoadjuvant or adjuvant chemotherapy in a preclinical model of resectable breast cancer. *Sci. Rep*. **6**, 36694; doi: 10.1038/srep36694 (2016).

**Publisher’s note:** Springer Nature remains neutral with regard to jurisdictional claims in published maps and institutional affiliations.

## Supplementary Material

Supplementary Information
