# Supplementary material for: Aflibercept and Ang1 supplementation improve neoadjuvant or adjuvant chemotherapy in a preclinical model of resectable breast cancer
Source: Sci Rep. 2016 Nov 14;6:36694. doi: 10.1038/srep36694 (PMC5107907; doi:10.1038/srep36694)
Supplement: Supplementary Information [file srep36694-s1.pdf]

SUPPLEMENT FOR:

Aflibercept and Ang1 supplementation improve neoadjuvant or adjuvant chemotherapy in a preclinical model of resectable breast cancer

Florence T.H. Wu[1,2], Marta Paez-Ribes[2], Ping Xu[2], Shan Man[2], Elena Bogdanovic[2], Gavin Thurston[3] and Robert S. Kerbel[1,2]*

[1]Department of Medical Biophysics, University of Toronto, Toronto, Ontario, Canada.

[2]Biological Sciences Platform, Sunnybrook Research Institute, Toronto, Ontario, Canada.

[3]Regeneron Pharmaceuticals, Inc., Tarrytown, New York, USA.

*To whom correspondence should be addressed: robert.kerbel@sri.utoronto.ca

---

Table of Contents | Page

Supplemental Figure S1. Aflibercept, with or without paclitaxel, is more effective than Ang/Tie2-targeted agents at suppressing LM2-4 primary tumor growth, as measured by volume ... 2

Supplemental Figure S2. COMP-Ang1 as primary tumor therapy and perioperative therapy ... 3

Supplemental Figure S3. Pulmonary micrometastases in mice bearing primary LM2-4 tumors, 29 days post-implantation. ... 4

Supplemental Table S1. Phase III Randomized Clinical Trials of Bevacizumab Plus Chemotherapy for Locally Recurrent or Metastatic Breast Cancer (mBC): Survival Outcomes ... 6

Supplemental Table S2. Phase III Clinical Trials of Neoadjuvant Bevacizumab Plus Chemotherapy for HER2-negative Early Breast Cancer: Pathological Complete Response (pCR) ... 7

Supplemental Table S3. Phase III Clinical Trials of Neoadjuvant and/or Adjuvant Bevacizumab Plus Chemotherapy for Early Breast Cancer: Survival Outcomes ... 8

Supplemental Table S4. Necropsy end-point data on disease distribution from the adjuvant therapy experiment shown in Figure 3 ... 9

Supplemental Table S5. Necropsy end-point data on disease distribution from the adjuvant therapy experiment shown in Figure 4 ... 9

Supplemental Methods: COMP-Ang1 production and dosing ... 10

Supplemental References ... 11

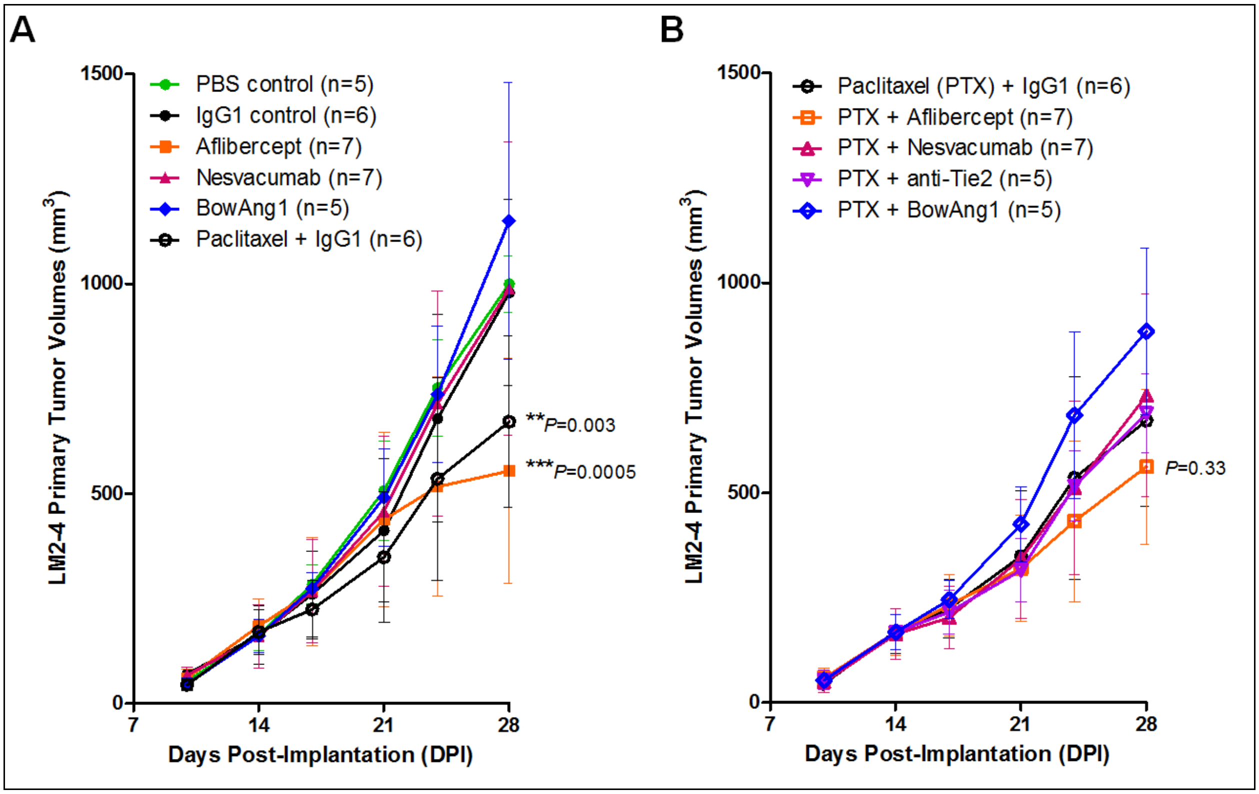

<u>Supplemental Figure S1</u>. **Aflibercept, with or without paclitaxel, is more effective than Ang/Tie2-targeted agents at suppressing LM2-4 primary tumor growth, as measured by volume.** 14 days after orthotopic implantation of 2×10^6 LM2-4 cells, mice bearing primary breast tumors averaging 164 mm^3 were randomized and treated for 2 weeks with the controls (PBS vehicle or IgG1 isotype), aflibercept (anti-VEGF-A/VEGF-B/PlGF), nesvacumab (anti-Ang2), BowAng1, or an anti-Tie2 antibody, with or without paclitaxel chemotherapy. Monotherapy groups (A) and combination treatment groups (B) were all from the same experiment and only displayed in separate panels for clarity. Mean tumor volumes ± SEM are depicted over time. Only the end-point tumor volumes were subjected to statistical analysis by two-sampled unpaired t tests (n = 5 to 11) for predefined comparisons. In (A), compared to untreated controls, the only monotherapies that significantly reduced terminal tumor volume were aflibercept (****P*=0.0005; with a difference between means of 434 mm^3 (95% CI of 220 to 648 mm^3)) and paclitaxel (***P*=0.003; with a difference between means of 317 mm^3 (95% CI of 124 to 509 mm^3)). In (B), compared to paclitaxel alone, the addition of aflibercept did not lead to a statistically significant further reduction in mean terminal tumor volume (*P*=0.33).

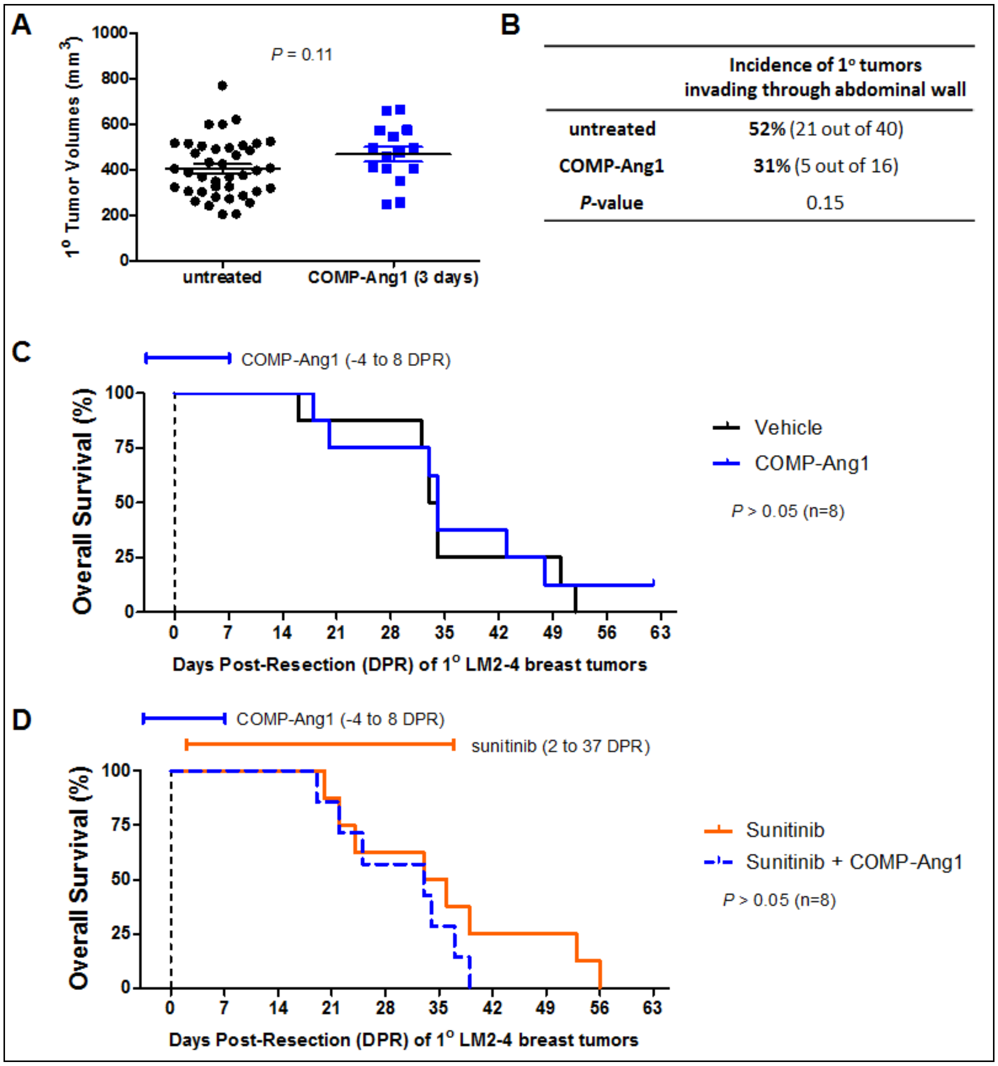

Supplemental Figure S2. COMP-Ang1 as primary tumor therapy and perioperative therapy. Orthotopic primary breast tumors averaging 400mm^3 were surgically resected around 21 days after implantation of 2×10^6 LM2-4 cells in the right inguinal mammary fat pad of SCID mice. COMP-Ang1 was given as a 12-day perioperative therapy, beginning four day before surgery (80µg every 3 days, IP; 4 doses in total). Sunitinib – an antiangiogenic tyrosine kinase inhibitor whose targets include VEGFR2 – was given as a 5-week adjuvant therapy, starting two days after surgery (60mg/kg daily, by oral gavage). **A:** Primary breast tumor volumes after 3 days of vehicle vs. pre-operative COMP-Ang1 treatment, compared by a *t* test. Means ± SEM are depicted. **B:** χ^2 test for association between 4 days of pre-operative COMP-Ang1 treatment and the proportion of primary breast tumors invading through the abdominal wall as detected by gross examination during surgical resection. **C-D:** Kaplan-Meier analyses of overall survival (OS): 12-day perioperative COMP-Ang1 therapy did not significantly prolong OS (*P*>0.05) when given as a monotherapy (**C**) or when given concurrently with adjuvant sunitinib therapy (**D**).

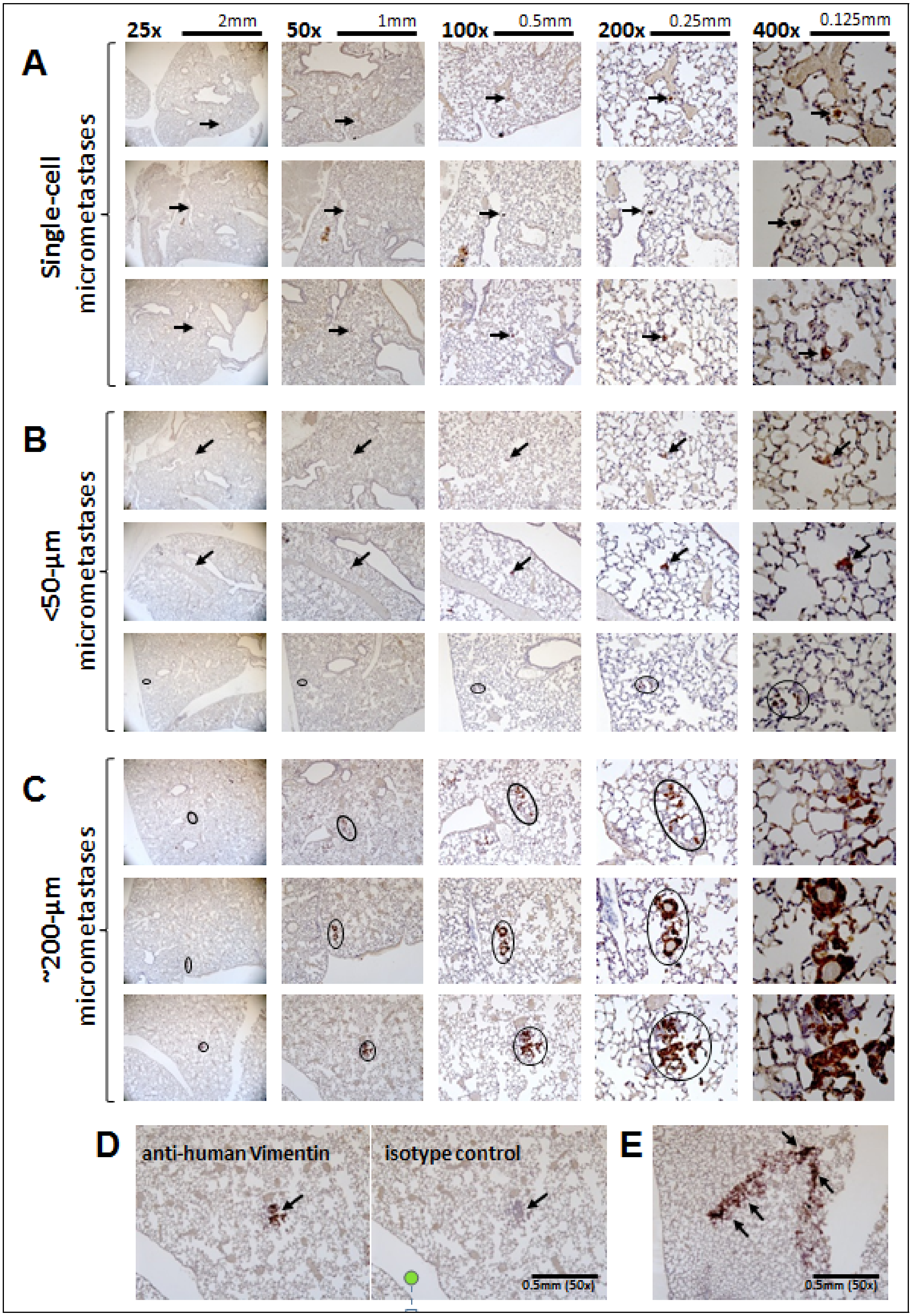
25x
2mm
50x
1mm
100x
0.5mm
200x
0.25mm
400x
0.125mm
A
Single-cell micrometastases
B
<50-µm micrometastases
C
~200-µm micrometastases
D
anti-human Vimentin
isotype control
0.5mm (50x)
E
0.5mm (50x)

__Supplemental Figure S3__**. Pulmonary micrometastases in mice bearing primary LM2-4 tumors, 29 days post-implantation.** Metastatic nodules in cross-sections of mouse lungs, as identified by IHC staining for human vimentin, were very sparse at the endpoint of this experiment. When present, lung micrometastases consisted of mostly single cells (**A**) or tiny <50-µm clusters (**B**). On rare occasions, slightly larger ~200-µm micrometastases (**C**) could be observed. The specificity of the staining antibody is demonstrated in (**D**). Given the rarity of lung micrometastases (low signal), they could not be reliably distinguished from background noise (e.g., from tissue folding as shown in (**E**)) through automated image processing and thus were not quantified.

Supplemental Table S1. Phase III Randomized Clinical Trials of Bevacizumab Plus Chemotherapy for Locally Recurrent or Metastatic Breast Cancer (mBC): Survival Outcomes

Trial	Setting	Arms	Median PFS (months)	Median OS (months)
**AVF2119** [1] (open-label)	2nd-line therapy for mBC	Capecitabine (1,250 mg/m^2, bid 14/21)	4.2	14.5
		Capecitabine + **Bevacizumab (15mg/kg, q3w)**	4.9 (HR=0.98, *P*=0.86)	15.1 (*P*>0.05)
**E2100** [2] (open-label)	1st-line therapy for mBC	Paclitaxel (90mg/m^2, qw 3/4)	5.9	25.2
		Paclitaxel **+ Bevacizumab (10mg/kg, q2w)**	11.8 (HR=0.60, *P*<0.001)	26.7 (HR=0.88, *P*=0.16)
**AVADO**[†] [3] (double-blind)	1st-line therapy for HER2$^-$ mBC	Docetaxel (100mg/m^2, q3w) + Placebo	8.1	31.9
		Docetaxel **+ Bevacizumab (7.5mg/kg, q3w)**	9.0 (stratified HR=0.80, *P*=0.045)	20.8 (HR=1.05, *P*=0.72)
		Docetaxel + **Bevacizumab (15mg/kg, q3w)**	10.1 (stratified HR=0.67, *P*<0.001)	30.2 (HR=1.03, *P*=0.85)
**RIBBON-1**[†] [4] (double-blind)	1st-line therapy for HER2$^-$ mBC	Capecitabine (1,000 mg/m^2, bid 14/21) + Placebo	5.7	
		Capecitabine + **Bevacizumab (15mg/kg, q3w)**	8.6 (HR=0.69, *P*<0.001)	N/A (HR=0.85, *P*=0.27)
		Taxane/Anthracycline[‡] + Placebo	8.0	
		Taxane/Anthracycline[‡] + **Bevacizumab (15mg/kg, q3w)**	9.2 (HR=0.64, *P*<0.001)	N/A (HR=1.03, *P*=0.83)
**RIBBON-2**[†5] (double-blind)	2nd-line therapy for HER2$^-$ mBC (Bev-naïve)	Chemotherapy (capecitabine/ taxane/gemcitabine/vinorelbine) + Placebo	5.1	16.4
		Chemotherapy + **Bevacizumab (10mg/kg q2w or 15mg/kg q3w)**	7.2 (stratified HR=0.78, *P*=0.007)	18.0 (stratified HR=0.90, *P*=0.37)
**TANIA** [6] (open-label)	2nd-line therapy for HER2$^-$ mBC (after 1st-line Bev+Chemo)	Chemotherapy (capecitabine/ taxane/anthracycline/vinorelbine/ gemcitabine/cyclophosphamide) + Placebo	4.2	N/A (pending)
		Chemotherapy + **Bevacizumab (10mg/kg q2w or 15mg/kg q3w)**	6.3 (stratified HR=0.75, *P*=0.007)	
**MERiDiAN** [7] (double-blind)	1st-line therapy for HER2$^-$ mBC	Paclitaxel (90mg/m^2, qw 3/4) + Placebo	8.8	N/A (pending)
		Paclitaxel **+ Bevacizumab (10mg/kg, q2w)**	11.0 (HR=0.68, *P*=0.0007)	

HR = hazard ratio. **PFS** = progression-free survival. **OS** = overall survival. [‡]Docetaxel, nab-paclitaxel, or doxorubicin-cyclophosphamide, epirubicin-cyclophosphamide, fluorouracil-epirubicin-cyclophosphamide, or fluorouracil-doxorubicin-cyclophosphamide. [†]Cross-over design [8].

Supplemental Table S2. Phase III Clinical Trials of Neoadjuvant Bevacizumab Plus Chemotherapy for HER2-negative Early Breast Cancer: Pathological Complete Response (pCR)

Trial & Arms	pCR, overall	pCR, best subgroup	Definition of pCR
**GeparQuinto/GBG-44** [9]	**HER2$^-$** (*P*=0.04)	**TNBC** (*P*=0.003)	**ypT0 ypN0** (absence of invasive and intraductal disease in breast & axillary lymph nodes)
**Bevacizumab**+EC→T	18.4%	39.3%	
EC→T	14.9%	27.3%	
**NSABP-B40** [10]	**HER2$^-$** (*P*=0.02)	**HER2$^-$ HR$^+$** (*P*=0.007)	**ypT0/is ypNx** (absence of invasive disease in breast only)
**Bevacizumab**+T(X/G)→AC	34.5%	23.2%	
T(X/G)→AC	28.2%	15.1%	
**ARTemis** [11]	**HER2$^-$** (*P*=0.03)	**HER2$^-$ ER$^-$** (*P*=N/A)	**ypT0/is ypN0** (absence of invasive disease in breast or axillary lymph nodes)
**Bevacizumab**+D→FEC	22%	45%	
D→FEC	17%	31%	

All three trials were open-label and randomized. Bevacizumab was given 15mg/kg, q3w, IV in all three trials. **pCR** = pathological complete response. **HER2** = human epidermal growth factor receptor 2, also known as Erbb2 or Neu. **ER** = estrogen receptor. **PgR** = progesterone receptor. **HR$^+$** = hormone receptor-positive (ER$^+$ and/or PgR$^+$). **TNBC** = triple-negative breast cancer (HER2$^-$, ER$^-$ and PgR$^-$). **EC→T** = epirubicin-cyclophosphamide followed by docetaxel. **T(X/G)→AC** = docetaxel-(capecitabine/gemcitabine) followed by doxorubicin-cyclophosphamide. **D→FEC** = docetaxel followed by fluororuacil-epirubicin-cyclophosphamide.

Supplemental Table S3. Phase III Clinical Trials of Neoadjuvant and/or Adjuvant Bevacizumab Plus Chemotherapy for Early Breast Cancer: Survival Outcomes

Trial & Arms	Disease-Free Survival (DFS)		Overall Survival (OS)	
**GeparQuinto/GBG-44 (HER2^{-} BC)** [6]	**3-year DFS, Overall** HR=1.03, *P*=0.784	**3-year DFS, TNBC** HR=0.99, *P*=0.941	**3-year OS, Overall** HR=0.97, *P*=0.842	**3-year OS, TNBC** HR=1.02, *P*=0.891
Neoadjuvant {EC**Bev**→T**Bev**}	78.9%	N/A	87.9%	N/A
Neoadjuvant {EC→T}	79.9%	N/A	87.8%	N/A
**ARTemis (HER2^{-} BC)** [12]	**3-year DFS** HR=0.86, *P*=0.29		**3-year OS** HR=0.80, *P*=0.19	
Neoadjuvant {**Bev+**D→FEC}	77%		85%	
Neoadjuvant {D→FEC}	80%		87%	
**NSABP (HER2^{-} BC)** [13]	**5-year DFS** HR=0.80, *P*=0.06		**5-year OS** HR=0.65, *P*=0.004	
Neoadjuvant {T(X/G)**Bev**→AC**Bev**} + Adjuvant {**Bev**}	77.0%		85.8%	
Neoadjuvant {T(X/G)→AC}	72.7%		80.6%	
**BEATRICE (TNBC)** [14]	**3-year invasive DFS (IDFS)** HR=0.88, *P*=0.18		**3-year OS** HR=0.84, *P*=0.23	
Adjuvant {A/T**Bev** →**Bev** }	83.7%		93%	
Adjuvant {A/T}	82.7%		92%	
**ECOG-5103 (HER2^{-} BC)** [15]	**5-year invasive DFS (IDFS)** HR=0.87, *P*=0.17		**5-year OS** HR=0.89, *P*=0.41	
Adjuvant {AC**Bev**→T**Bev**→**Bev**}	80%		N/A	
Adjuvant { AC→T}	77%		N/A	
**BETH (HER2^{+} BC)** [16]	**3-year invasive DFS (IDFS)** HR=1.00, *P*=0.98		**3-year OS** HR=0.87, *P*=0.4387	
Adjuvant {TCH**Bev**→H**Bev**}, or Adjuvant{TH**Bev**→FEC→H**Bev**}	92%		97%	
Adjuvant {TCH→H}, or Adjuvant {TH→FEC→H}	92%		96%	

All trials were open-label and randomized. **HER2** = human epidermal growth factor receptor 2, also known as Erbb2 or Neu. **ER** = estrogen receptor. **PgR** = progesterone receptor. **HR^{+}** = hormone receptor-positive (ER^{+} and/or PgR^{+}). **TNBC** = triple-negative breast cancer (HER2^{-}, ER^{-} and PgR^{-}). **Bev** = bevacizumab at dose equivalent of 5mg/kg/wk (i.e., 10mg/kg q2w or 15mg/kg q3w). **EC→T** = epirubicin-cyclophosphamide followed by docetaxel. **D→FEC** = docetaxel followed by fluororuacil-epirubicin-cyclophosphamide. **T(X/G)→AC** = docetaxel-(capecitabine/gemcitabine) followed by doxorubicin-cyclophosphamide. **A/T** = anthracycline, taxane, or both. **AC→T** = doxorubicin-cyclophosphamide followed by docetaxel. **TCH** = docetaxel-carboplatin-trastuzumab followed by trastuzumab. **TH→FEC→H** = docetaxel-trastuzumab followed by 5-fluorouracil-epirubicin-cyclophosphamide followed by trastuzumab.

Supplemental Table S4. Necropsy end-point data on disease distribution from the adjuvant therapy experiment shown in Figure 3.

Group	n	Local tumor regrowth at primary site		Ascites	Lung metastases	Distant lymph node metastases	Limb paralysis/ impairment
		All	Invasive				
Vehicle	9	56%	56%	22%	67%	67%	22%
Aflibercept	9	33%	33%	33%	78%	44%	33%
Nesvacumab	7	100%	86%	29%	71%	57%	29%
BowAng1	8	63%	38%	50%	63%	63%	25%
Paclitaxel (PTX)	9	56%	22%	22%	89%	67%	22%
PTX+Aflibercept	8	50%	25%	25%	88%	50%	25%
PTX+Nesvacumab	8	38%	38%	13%	75%	38%	38%
PTX+antiTie2	8	38%	38%	25%	75%	88%	25%
PTX+BowAng1	8	38%	0%	0%	75%	63%	25%
All groups:	74	51%	36%	24%	76%	59%	27%

Supplemental Table S5. Necropsy end-point data on disease distribution from the adjuvant therapy experiment shown in Figure 4.

Group	n	Local tumor regrowth at primary site (all non-invasive)	Abdominal Tumor Burden or Ascites	Lung metastases	Distant lymph node metastases	Limb paralysis/ impairment
Paclitaxel (PTX)	10	2	1	7	5	4
PTX+Aflibercept	10	3	0	7	5	4
PTX+BowAng1	10	0	2	8	7	3
PTX+Aflibercept +BowAng1	10	2	1	6	3	3
All groups:	40	18%	10%	70%	50%	35%

SUPPLEMENTAL METHODS

COMP-Ang1 production and dosing

COMP-Ang1 was produced in the laboratory of Dr. RS Kerbel using a stable CHO cell line engineered to express FLAG-tagged COMP-Ang1 provided by Dr. GY Koh [17]. CHO cells were grown in suspension culture in serum-free media (Hyclone #SH30549), with 500nM of methotrexate (Sigma #06563), on a shaker set at 120 rpm in a humidified incubator (37°C, 5% CO_2, 21% O_2), maintained between 2x10^5 to 1x10^6 cells/mL at > 90% cell viability. COMP-Ang1 was then purified from the harvested and 0.2 µm-filtered culture media by gravity column chromatography, using anti-FLAG M2 affinity gel resin (Sigma #A2220) for immunoprecipitation and 100 µg/mL FLAG peptide (Sigma #F3290) in PBS with 0.1% CHAPS for competitive elution. Elutes were then dialyzed through a centrifugal concentrator (10,000 MWCO, Millipore). COMP-Ang1 purity was confirmed by SDS-PAGE and Coomassie Brilliant Blue (R-250) staining. Protein identity and pentameric structure was confirmed by SDS-PAGE (under reducing vs. non-reducing conditions) and immunoblotting with an antibody to Ang1 (R&D Systems #AF923). Protein concentration was determined by a BCA protein assay (Pierce). Functional binding to Tie2 was confirmed by surface plasmon resonance (Biacore T200) as previously described [18]. Purified COMP-Ang1 was diluted in PBS to 100µg/200µL for *in vivo* IP injections.

References

1. Miller, K. D. *et al*. Randomized phase III trial of capecitabine compared with bevacizumab plus capecitabine in patients with previously treated metastatic breast cancer. *J. Clin. Oncol.* **23**, 792-799 (2005).

2. Miller, K. *et al*. Paclitaxel plus bevacizumab versus paclitaxel alone for metastatic breast cancer. *N. Engl. J. Med.* **357**, 2666-2676 (2007).

3. Miles, D. W. *et al*. Phase III study of bevacizumab plus docetaxel compared with placebo plus docetaxel for the first-line treatment of human epidermal growth factor receptor 2-negative metastatic breast cancer. *J. Clin. Oncol.* **28**, 3239-3247 (2010).

4. Robert, N. J. *et al*. RIBBON-1: randomized, double-blind, placebo-controlled, phase III trial of chemotherapy with or without bevacizumab for first-line treatment of human epidermal growth factor receptor 2-negative, locally recurrent or metastatic breast cancer. *J. Clin. Oncol.* **29**, 1252-1260 (2011).

5. Brufsky, A. M. *et al*. RIBBON-2: a randomized, double-blind, placebo-controlled, phase III trial evaluating the efficacy and safety of bevacizumab in combination with chemotherapy for second-line treatment of human epidermal growth factor receptor 2-negative metastatic breast cancer. *J. Clin. Oncol.* **29**, 4286-4293 (2011).

6. von Minckwitz, G. *et al*. Bevacizumab plus chemotherapy versus chemotherapy alone as second-line treatment for patients with HER2-negative locally recurrent or metastatic breast cancer after first-line treatment with bevacizumab plus chemotherapy (TANIA): an open-label, randomised phase 3 trial. *Lancet Oncol.* **15**, 1269-1278 (2014).

7. Miles, D. *et al*. First results from the double-blind placebo (PL)-controlled randomised phase III MERiDiAN trial prospectively evaluating plasma (p)VEGF-A in patients (pts) receiving first-line paclitaxel (PAC) +/- bevacizumab (BV) for HER2-negative metastatic breast cancer (mBC). [Abstract #1866] In: 2015 European Cancer Congress annual meeting. (2015).

8. Gonzalez-Angulo, A. M., Hortobagyi, G. N. & Ellis, L. M. Targeted therapies: peaking beneath the surface of recent bevacizumab trials. *Nat. Rev. Clin. Oncol.* **8**, 319-320 (2011).

9. von Minckwitz, G. *et al*. Neoadjuvant chemotherapy and bevacizumab for HER2-negative breast cancer. *N. Engl. J. Med.* **366**, 299-309 (2012).

10. Bear, H. D. *et al*. Bevacizumab added to neoadjuvant chemotherapy for breast cancer. *N. Engl. J. Med.* **366**, 310-320 (2012).

11. Earl, H. M. *et al*. Efficacy of neoadjuvant bevacizumab added to docetaxel followed by fluorouracil, epirubicin, and cyclophosphamide, for women with HER2-negative early breast cancer (ARTemis): an open-label, randomised, phase 3 trial. *Lancet Oncol.* **16**, 656-666 (2015).

12. Earl, H. M. *et al*. Disease-free (DFS) and overall survival (OS) at 3.4 years (yrs) for neoadjuvant bevacizumab (Bev) added to docetaxel followed by fluorouracil, epirubicin and cyclophosphamide (D-FEC), for women with HER2 negative early breast cancer: The ARTemis trial. [Abstract] In: 2016 ASCO Annual Meeting. *J Clin Oncol ,* **34 suppl**, (abstr 1014) (2016).

13. Bear, H. D. *et al*. Neoadjuvant plus adjuvant bevacizumab in early breast cancer (NSABP B-40 [NRG Oncology]): secondary outcomes of a phase 3, randomised controlled trial. *Lancet Oncol.* **16**, 1037-1048 (2015).

14. Cameron, D. *et al*. Adjuvant bevacizumab-containing therapy in triple-negative breast cancer (BEATRICE): primary results of a randomised, phase 3 trial. *Lancet Oncol.* **14**, 933-942 (2013).

15. Miller, K. *et al*. Bevacizumab (Bv) in the adjuvant treatment of HER2-negative breast cancer: Final results from Eastern Cooperative Oncology Group E5103. [Abstract] In: 2014 ASCO Annual Meeting. *J Clin Oncol* **32:5s suppl.**, (abstr 500) (2014).

16. Slamon, D. J. *et al*. Primary results from BETH, a phase 3 controlled study of adjuvant chemotherapy and trastuzumab ± bevacizumab in patients with HER2-positive, node-positive or high risk node-negative breast cancer. [Abstract] In: 2013 San Antonio Breast Cancer Symposium. *Cancer Res* **73**, (abstr S1–03) (2013).

17. Hwang, S. J. *et al*. High-level expression and purification of a designed angiopoietin-1 chimeric protein, COMP-Ang1, produced in Chinese hamster ovary cells. *Protein J.* **27**, 319-326 (2008).

18. Wu, F. T. *et al*. Vasculotide reduces endothelial permeability and tumor cell extravasation in the absence of binding to or agonistic activation of Tie2. *EMBO Mol. Med.* **7**, 770-787 (2015).
